# Supplementary material for: Tead1a Initiates Transcriptional Priming Through the TEAD1a/YAP‐Notch1‐Spi1/Cebpα Axis to Promote Neutrophil Fate
Source: Adv Sci (Weinh). 2025 Aug 28;12(41):e05441. doi: 10.1002/advs.202505441 (PMC12591127; doi:10.1002/advs.202505441)
Supplement: Supplementary file 4 — Supplementary Dataset [file ADVS-12-e05441-s004.zip › 3-1.zip!/3-1/02-1.cluster/cluster2/Enrich/ego_MF.pdf]

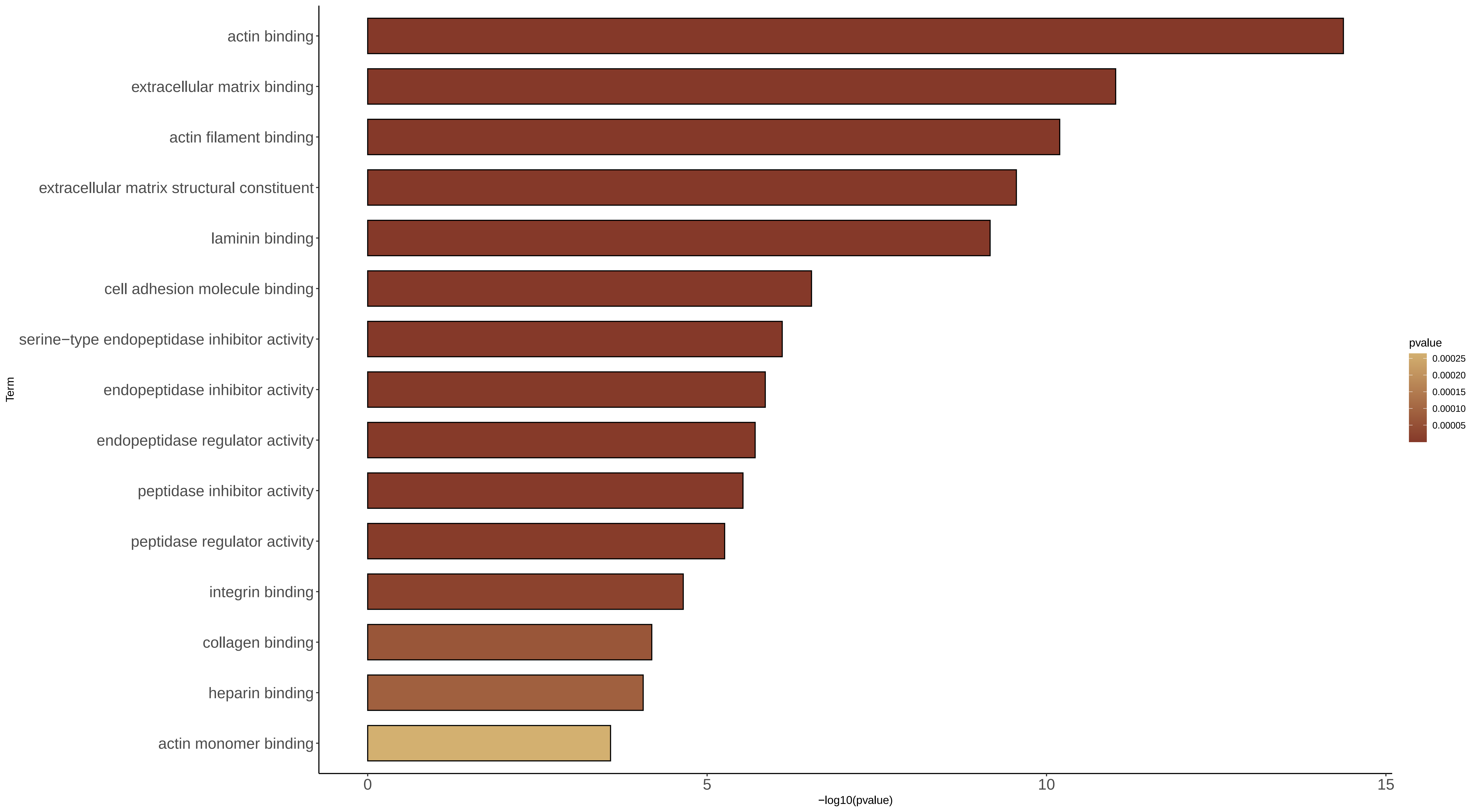
actin binding
extracellular matrix binding
actin filament binding
extracellular matrix structural constituent
laminin binding
cell adhesion molecule binding
serine−type endopeptidase inhibitor activity
endopeptidase inhibitor activity
endopeptidase regulator activity
peptidase inhibitor activity
peptidase regulator activity
integrin binding
collagen binding
heparin binding
actin monomer binding
Term
0
5
10
15
−log10(pvalue)
pvalue
0.00025
0.00020
0.00015
0.00010
0.00005
